# Plasmonic refractive index sensing using strongly coupled metal nanoantennas: nonlocal limitations

**DOI:** 10.1038/s41598-018-28011-x

**Published:** 2018-06-25

**Authors:** Hancong Wang

**Affiliations:** The Fujian Provincial Key Laboratory of Automotive Electronics and Electric Drive, Research center for Microelectronics Technology, School of Information Science and Engineering, Fujian University of Technology, Fuzhou, 350108 PR China

## Abstract

Localized surface plasmon resonance based on coupled metallic nanoparticles has been extensively studied in the refractive index sensing and the detection of molecules. The amount of resonance peak-shift depends on the refractive index of surrounding medium and the geometry/symmetry of plasmonic oligomers. It has recently been found that as the feature size or the gap distance of plasmonic nanostructures approaches several nanometers, quantum effects can change the plasmon coupling in nanoparticles. However, most of the research on plasmonic sensing has been done based on classical local calculations even for the interparticle gap below ~3 nm, in which the nonlocal screening plays an important role. Here, we theoretically investigate the nonlocal effect on the evolution of various plasmon resonance modes in strongly coupled nanoparticle dimer and trimer antennas with the gap down to 1 nm. Then, the refractive index sensing in these nonlocal systems is evaluated and compared with the results in classical calculations. We find that in the nonlocal regime, both refractive index sensibility factor and figure of merit are actually smaller than their classical counterparts mainly due to the saturation of plasmon shifts. These results would be beneficial for the understanding of interaction between light and nonlocal plasmonic nanostructures and the development of plasmonic devices such as nanosensors and nanoantennas.

## Introduction

Surface plasmons (SP) is the collective electromagnetic (EM) excitation of conduction electrons at metal-dielectric boundary, which can induce the confinement and enhancement of electric field near the surface of noble metallic nanostructures^1^. Local SP have been exploited to manipulate light-related phenomena at the nanoscale^2,3^, e.g. near-field enhancement (or “hot spot” distribution)^4–6^, emission polarization^7–10^, scattering direction^11^, optical force^12,13^, spin-orbit interaction^14^, and charge transfer^15^. Many of these manipulations could be used for plasmonic sensing^16–19^, stemmed from the effect of the dielectric function (or refractive index) of surrounding medium on the surface plasmon resonance (SPR)^20^. The sensing techniques using SP include: (i) surface-enhanced spectroscopys based on the amplification of the optical field at the gaps of few nanometers^4,19,21,22^; (ii) SPR sensors^16,17^ including surface plasmon polaritons (SPP) sensors and localized surface plasmon resonance (LSPR) sensors^23^. Among them, the LSPR sensor has the advantage of portability involving individual plasmonic nanoparticles suspended in solutions or supported on substrates^24,25^. For a nanoscale volume of chemical/biological species adsorbed on the metal nanostructures, although the contrast in refractive index (RI) is tiny, it is still possible to induce a measurable shift of resonant peak positions^26,27^.

Actually, many factors may affect the real plasmonic sensing measured in experiments, such as, (i) the *absorption effect* in particles of large size^28^, (ii) the *near-field enhancement effect* because of atomistic modify of surface shape in solution due to such as the molecule functionalization^29,30^, and (iii) the *size distribution effect* of nanoparticle solutions that would broaden the plasmonic resonance and decrease the figure of merit (FoM). While for the strongly coupled metal nanoparticles, as the gap distance narrows, the classical EM theory predicts an ultrahigh near-field enhancement in the gap^31–34^, a diverging red shift of bonding plasmon resonance, and a strong far-field scattering. Recently, it was reported that plasmonic quantum effects, e.g. nonlocality (<3 nm) and electron tunneling (<1 nm), will emerge and drastically change the plasmonic responses for gaps approaching 1 nm^35–52^. For example, the nonlocality of the plasmon-induced screening charge might be sensitive to the refractive index of surrounding media^53^, especially for typical interparticle gap below ~3 nm scale (nonlocal regime)^9^. Hence, *nonlocal effect* on RI sensing should also be fully considered in strongly coupled metal nanoantennas.

In this work, we theoretically investigate the plasmon resonance evolutions and RI sensing of plasmonic nanoparticle dimer and trimer with the gap distance down to nonlocal regime. The resonance peak position and RI sensing as a function of gap and geometry/symmetry are evaluated by the generalized Mie theory (GMT)^54,55^ and local analogue model (LAM)^44,45^ incorporating nonlocality. In the classical regime, for the dimer, the dipolar coupling (longitudinal bonding dipole, LBDP) results in the strong dependence of resonant peak on the gap distance, while the transverse dipole mode (TDP) shows weakly dependence feature. For the trimer, the change of its symmetry will bring various evolutions of scattering peaks. As to the RI sensing, higher sensibility is found for the parallel polarized incidence. However, as the gap distance further narrows to 1~3 nm range, nonlocal effects manifests as a saturation of resonant red-shift due to the reduction of screening charge density in the gap, which reduces the RI sensibility and FoM. These results indicate the possibility of manipulating the scattering and sensing by nonlocal plasmons and developing plasmonic nanosensors and nanoantennas.

## Results

### Plasmon resonances of nanoantennas in nonlocal regime

**Figure 1:**
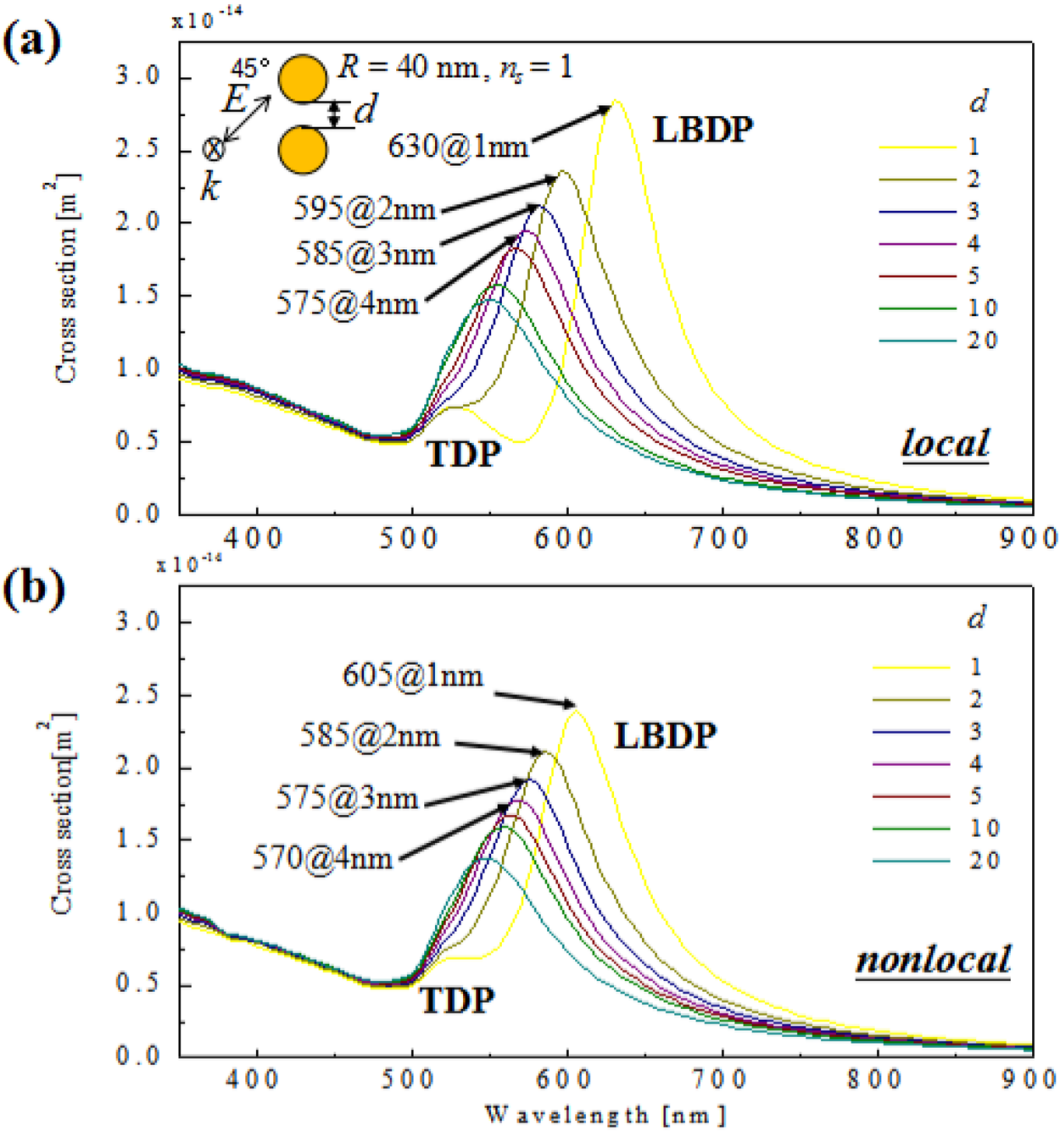
Scattering spectra of an individual spherical gold nanoparticle dimer with different gap distances (left to right, 20 nm to 1 nm) under a 45° polarized excitations. (**a**) Classical local prediction by GMT and (**b**) incorporating nonlocal effect by LAM. The radius of nanoparticle is R = 40 nm. Abbreviations for resonances: LBDP, longitudinal bonding dipole; TDP, transverse dipole.

Figure 1 shows the simulated scattering spectra of gold nanoparticle dimers with the interparticle gap distance decreasing from 20 nm to 1 nm. The radius of the gold sphere used in simulation is 40 nm. To mimic an unpolarized excitation, the polarization of normal incident plane wave was set as 45° relative to the longitudinal axis of dimer antennas. The RI of the surrounding medium is set to be 1. The classical local simulation shown in Fig. 1(a) is performed based on GMT (see methods for details). For large interparticle distances (10~20 nm), the EM coupling between nanoparticles is weak. In this situation, the LBDP mode generated by two nanoparticles interaction is close to the uncoupled TDP mode that mainly contributes from single nanoparticle dipolar resonance. Actually, the interaction energy between two nanoparticles can be expressed by: *V* ~ *p*_1*_p_2_*/d*^3^, where *p* indicates the dipole moments of a single nanoparticle and *d* is the gap distance^1^. Obviously, the interaction energy will dramatically increase with the decrease of distance. As shown in Fig. 1(a), the LBDP significantly red-shifts from 540 nm to 630 nm when the interparticle distance decreases from 20 nm to 1 nm, while the uncoupled TDP mode shows little dependence on the distance.

However, as the feature size approaches to ~1 nm scale, the screening charge smear out due to the finite Fermi wavelength of electrons. This spatial dispersion (nonlocality) has a large impact on the optical response^38^. The dielectric permittivity in nonlocal regime depends on both the frequency and electron wavenumber. This correction is important for very small gaps less than 3 nm. Hence, we further performed the simulation using local analogue model (LAM) to incorporate the nonlocality into the local EM framework (see Methods for details). The nonlocal LBDP in Fig. 1(b) obviously shows less red shift than local case in Fig. 1(a) when the distance decreases from 3 nm to 1 nm.

**Figure 2:**
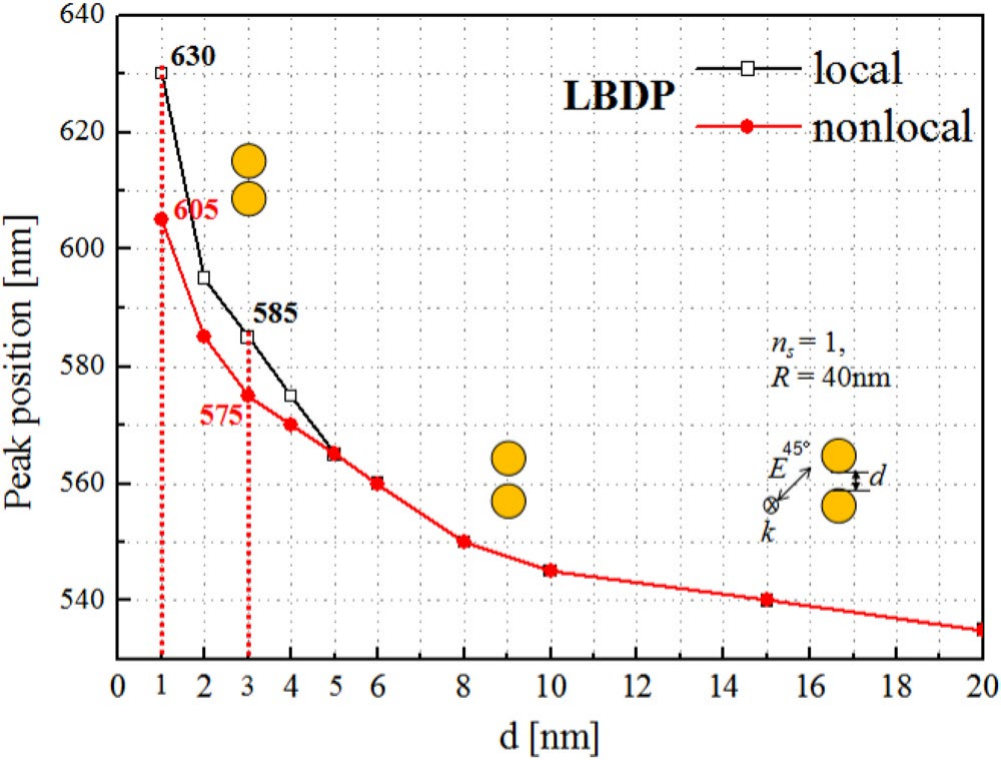
LBDP peak positions as a function of gap distances. Local prediction by GMT (black hollow squares) and results incorporating nonlocal effect by LAM (red solid circles). The lines are plotted to guide eyes.

Figure 2 summarizes the evolution of peak positions of LBDP modes as a function of the gap distance by local and nonlocal models. It is found that, when the distance is larger than ~3 nm, the nonlocal simulation almost agrees with the local one. However, as the distance is smaller than ~3 nm, the nonlocal result (red solid circles in Fig. 2) begins to deviate from the local prediction (black hollow squares in Fig. 2), showing less red shift than the local one. At *d* = 3 nm, the discrepancy of red shifts between the nonlocal and local cases is about 10 nm, which indicates that the nonlocal effect begins to play an important role. For a smaller gap, the discrepancy become larger. At *d* = 1 nm, the discrepancy is as large as 25 nm.

### Plasmonic sensing using strongly coupled dimer nanoantennas

**Figure 3:**
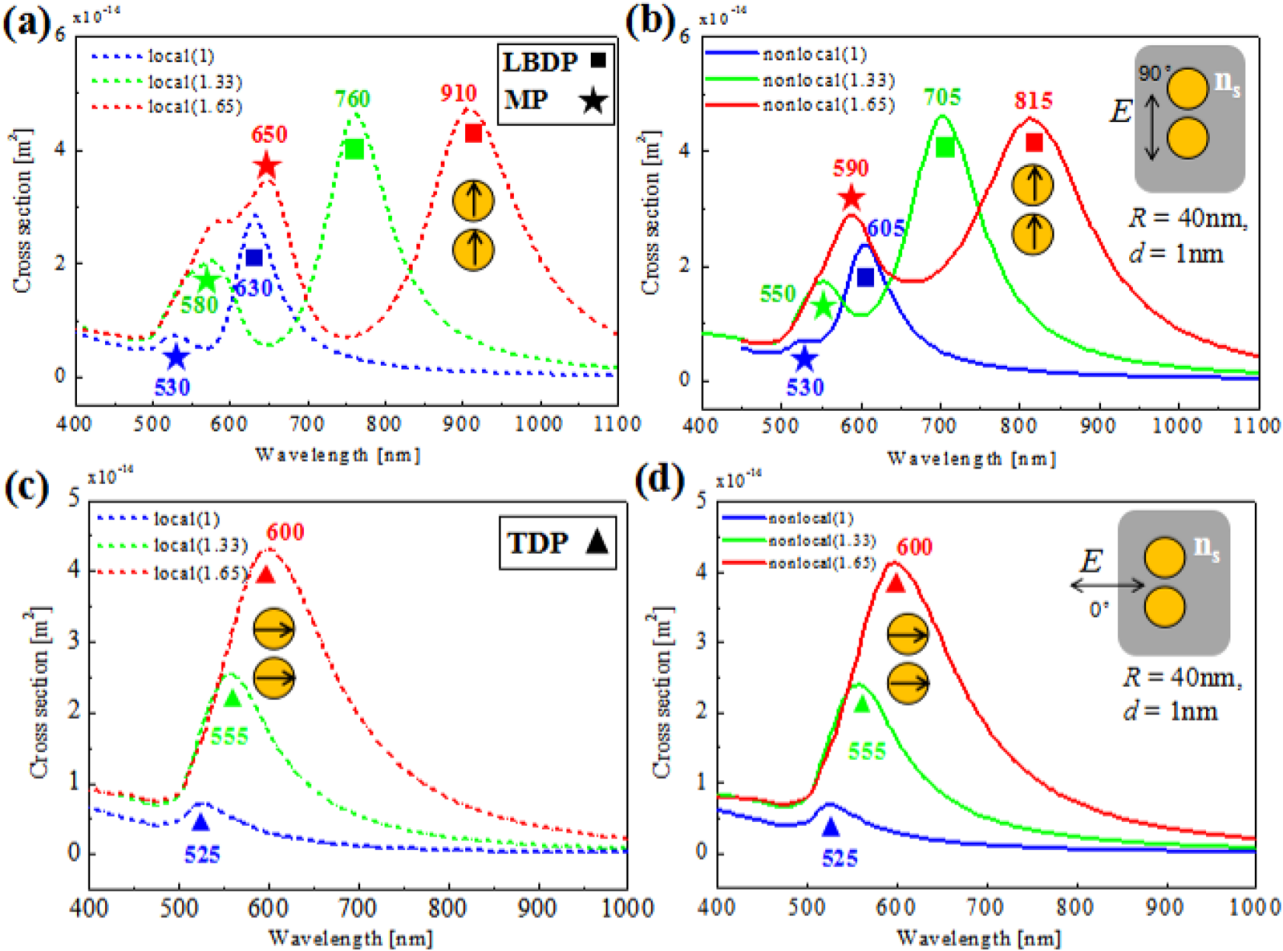
Scattering spectra of a dimer with different environmental refractive index *n*_*s*_. (**a**) Local and (**b**) nonlocal cases with parallel polarized excitation, respectively. (**c**) Local and (**d**) nonlocal cases with perpendicular polarized excitation, respectively. Blue, green, and red curves correspond to refractive index *n*_*s*_ = 1, 1.33, and 1.65, respectively.

Besides the gap distance, another important parameter influencing SP coupling is the dielectric environment^25^. Actually, the plasmonic sensitivity is strongly dependent on the size, shape, composition, and structure of nanostructrues^56^. Therefore, plasmonic nanoparticles are ideal platforms for refractive index sensing. In the following, we will discuss the nonlocal effect on the sensing properties of plasmonic nanoparticle dimer and trimers. Figure 3(a,b) show the simulated local and nonlocal scattering spectra of a gold dimer with the gap distance *d* = 1 nm under the parallel (90°) incident polarization, respectively. The environmental refractive index *n*_*s*_ = 1, 1.33, and 1.65 are represented by blue, green, and red curves, respectively. For local dimers in Fig. 3(a), the LBDP peak red-shifts significantly with the increasing of *n*_*s*_ due to the strong near-field plasmonic coupling between two nanoparticles under the parallel polarization. From *n*_*s*_ = 1 to 1.65, the LBDP mode almost linearly red-shifts from 630 nm to 910 nm. The multipole modes (MP) in shorter wavelength region can also be excited under the parallel polarized excitation. Different from the LBDP mode, the multipole mode slightly red-shifts from 530 nm to 650 nm. For the nonlocal dimers in Fig. 3(b), on the other hand, the magnitude of LBDP mode red shift is reduced. Correspondingly, from *n*_*s*_ = 1 to 1.65, the nonlocal LBDP mode red-shifts from 605 nm to 815 nm. While, the nonlocal effect has less influence on the multipole mode. The corresponding multipole peaks are from 530 nm to only 590 nm.

Then, we can analyze the sensing properties of dimers under the local and nonlocal cases. The resonance wavelength sensitivity factor *S* is usually used to quantify the performance of RI sensing, defined as the peak shift per unit change in the surrounding refractive index (RIU)^23^. This RI sensitivity factor for local LBDP is about *S* ≈ 431 nm/RIU that decrease to 323 nm/RIU for nonlocal case, which is about 25.1% reduction relative to the local prediction.

As to the peak width, in analogy to a damped oscillator, the peak width is directly related to its decay time, which in turn is determined by the damping of SP. The effect of peak width on sensing is typically evaluated by the FoM^24^, which is defined as the ratio of sensitivity factor *S* to scattering peak linewidth (FWHM, Full width at half maximum) *Δλ*, that is, FoM = *S/Δλ*. Actually, the modal linewidth can also be largely affected by nonlocality through surface-enhanced Landau damping, an effect that can be efficiently captured by the nonlocal optical response theory applied for instance in refs^35,48^, predicting drastic broadening of the plasmon modes. Here, for *n*_*s*_ = 1.33, the FoM of the dimer is about 3.6 and reduced to 2.7 if the nonlocal effect is considered. We find both the RI sensitivity factor and FoM are larger than their nonlocal counterparts due to more obvious plasmon shifts in local simulations. For the multipole modes, however, there are less difference between the local and nonlocal regime. Also, the angle of incident polarization dramatically changes the coupling and then the sensitivity of a dimer.

Figure 3(c,d) show the simulated local and nonlocal scattering spectra for the perpendicular (0°) incident polarization, respectively. Here, only the TDP mode can be excited. For TDP mode, its sensitivity factor *S* is 115 nm/RIU for both the local and nonlocal ones. As the RI sensitivity and nonlocality shows less influence on the uncoupled mode, we will mainly focus on the coupled modes in the trimer case.

### Plasmonic sensing using strongly coupled trimer nanoantennas

**Figure 4:**
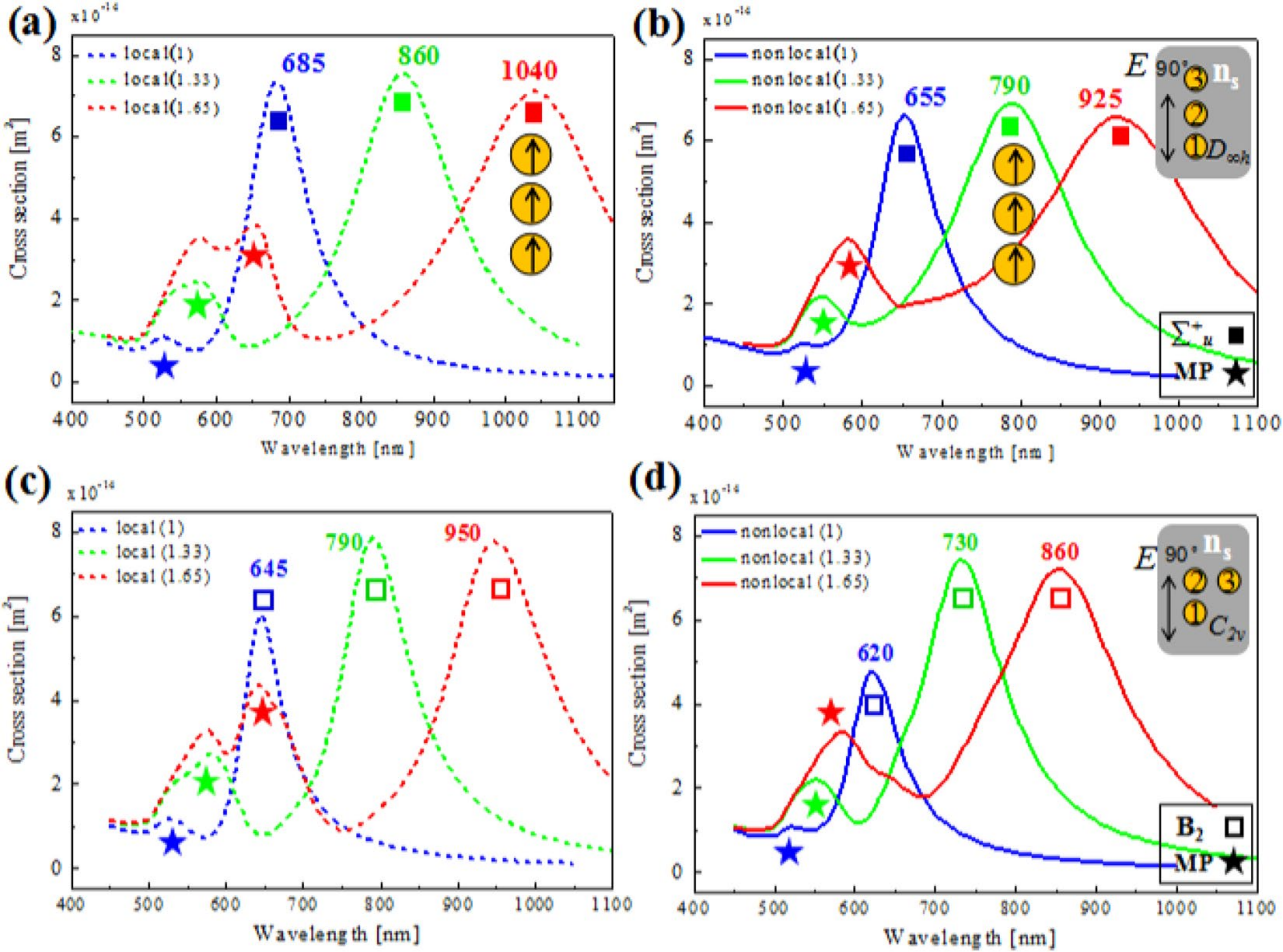
Scattering spectra of trimers with different environmental refractive index *n*_*s*_ under parallel polarization excitations. (**a**) Local and (**b**) nonlocal cases for linear timer, respectively. (**c**) Local and (**d**) nonlocal cases for right-angle trimer, respectively. Blue, green, and red curves correspond to refractive index *n*_*s*_ = 1, 1.33, and 1.65, respectively.

We consider two kinds of typical configurations, that is, linear trimer and right-angle (asymmetric) trimer. As shown in Fig. 4, the linear trimer (symmetry point group *D*_*∞h*_) or a right-angle trimer (group *C*_*2v*_) are formed by positioning a same particle on the top or right side of the 2^nd^ one^6,57–59^. Here, all the gaps between any two closely packed particles are 1 nm. Based on the plasmon hybridization (PH) theory^60^ and symmetry adopted linear combination (SALC) theory^57,58^, the main plasmon modes are ∑^+^_u_, ∏_u_ modes for *D*_*∞h*_ trimer, and B_2_, A_1_ modes for *C*_*2v*_ trimer. The linear trimer case with the polarization parallel to the trimer axis are shown in Fig. 4(a,b), where a strong coupled low-energy ∑^+^_u_ mode and a weak high-energy multipole mode are excited. In the local regime in Fig. 4(a), both modes red-shift as the increasing RI. From *n*_*s*_ = 1 to 1.65, the ∑^+^_u_ mode red-shifts from 685 nm to 1040 nm. The RI sensitivity factor of ∑^+^_u_ peak is about *S* ≈ 546 nm/RIU. This value is larger than the LBDP mode in dimer case. While for the nonlocal regime in Fig. 4(b), the ∑^+^_u_ mode red-shifts from 655 nm to 925 nm, corresponding to a RI sensitivity factor *S* ≈ 415 nm/RIU, which is about 24.0% reduction relative to the local prediction. The nonlocality caused-reduction of RI sensitivity in the linear trimer is slightly less than the dimer case. For *n*_*s*_ = 1.33, the FoM is about 3.2 (local) or 2.3 (nonlocal). Both RI sensitivity factor and FoM are smaller than their local cases due to the more obvious resonance shifts in classical simulations.

Figure 4(c,d) show the right-angle trimer (group *C*_*2v*_) with the polarization parallel to the dimer axis (1^st^–2^nd^). The strong coupled low-energy B_2_ mode and a weak high-energy multipole mode are excited. Similar to previous analysis, the classical local RI sensitivity for B_2_ mode is about 469 nm/RIU, which is reduced to 361 nm/RIU for the nonlocal case. For *n*_*s*_ = 1.33, the FoM is about 3.6 (local) or 2.6 (nonlocal). Because of its narrower width of resonance, the *C*_*2v*_ trimer has higher FoM than the case of *D*_*∞h*_ trimer.

## Discussion

**Figure 5:**
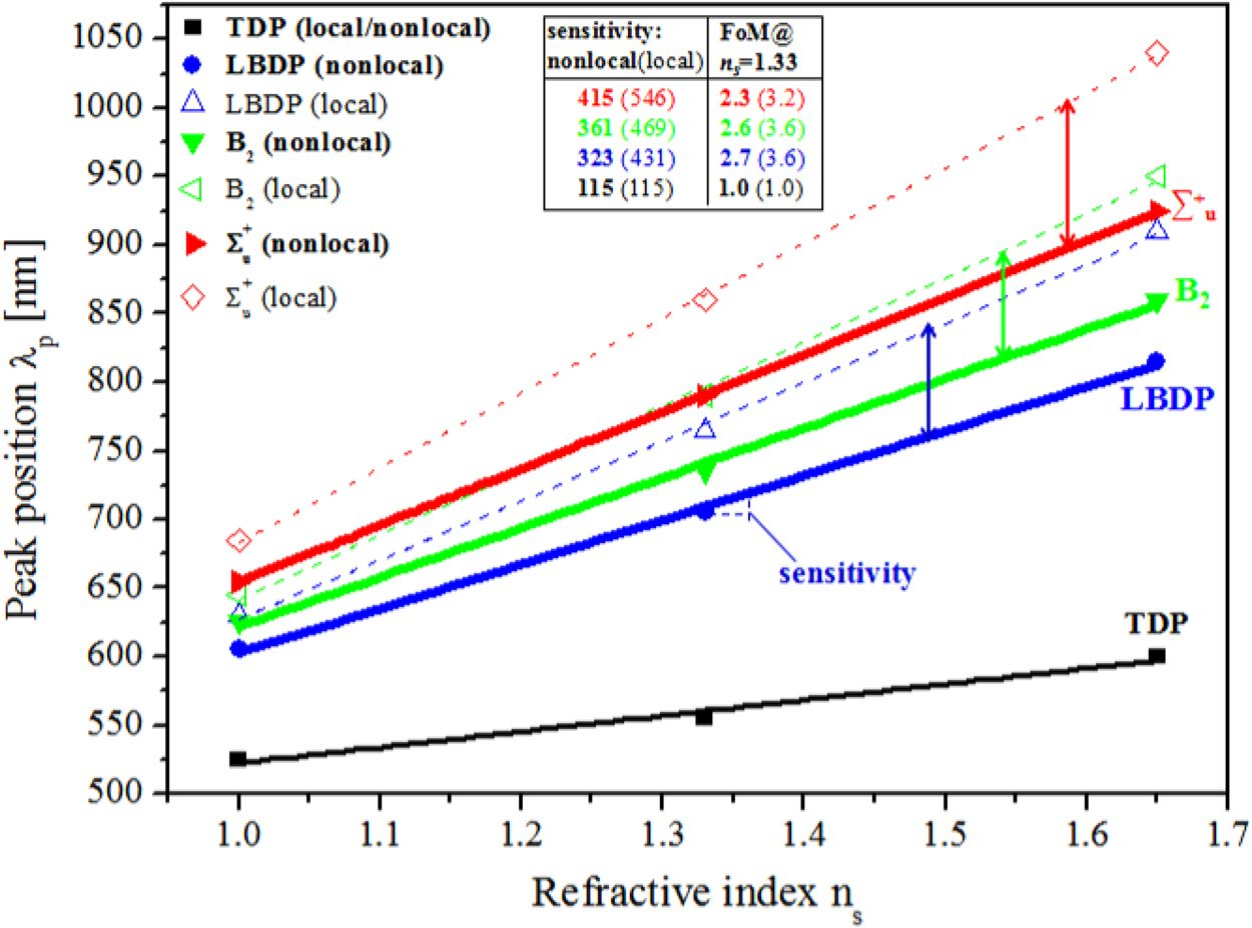
The nonlocal (solid lines) and local (dashed lines) resonance peak positions *λ*_*p*_ as a function of the environmental refractive index *n*_*s*_ for different modes. ∑^+^_u_ mode (red) in *D*_*∞h*_ trimer, B_2_ mode (green) in *C*_*2v*_ trimer, LBDP mode (blue) and TDP mode (black) in dimer. RI sensitivities (slope) are obtained using a linear fitting. The inset box lists RI sensitivities and FoMs calculated by nonlocal and local models. FoMs are calculated at *n*_*s*_ = 1.33.

To compare the RI sensitivities of different plasmon modes in nanoparticle antennas, we plot resonant peaks as a function of the environmental refractive index for four modes, including LBDP and TDP modes in dimer, ∑^+^_u_ modes in *D*_*∞h*_ trimer, and B_2_ modes in *C*_*2v*_ trimer. As shown in Fig. 5, the shift of all modes is approximately linear to the change of refractive index. The gradients for each line are the RI sensitivities which are summarized in the inset box in Fig. 5. Obviously, the nonlocal effect would reduce the RI sensitivity factor and FoM in all cases. Hence, for closed packed nanoparticles with the gap below ~3 nm, the nonlocal effect cannot be neglected.

In conclusion, we have investigated the scattering spectrum, RI sensitivity factor and FoM in plasmonic nanoparticle dimer and trimer antennas calculated within a local and a nonlocal framework. By comparing local and nonlocal results, it is shown that the classical local model works for the gap larger than 3 nm. As the gap further decreases to the nonlocal regime (<∼3 nm), the red shift of LBDP peak positions are not significant compared with local calculations. The evolution of various surface plasmon peaks is related to gap distance, the symmetry of plasmonic nanoparticles, and especially environmental refractive index. The scattering from plasmon modes in nanoparticles can be used for refractive index sensing. A higher RI sensibility and figure of merit are found in dimer for parallel polarized excitations due to strong plasmonic coupling. Because of less plasmonic shifts, both the RI sensitivity factor and FoM of dimer and trimer nanoantennas in the nonlocal regime are smaller than their local ones. These results somehow advance the understanding on the cross-over between local and nonlocal regimes, and would be beneficial for the study of nonlocal effect on the emission from nanoantennas and the development of plasmonic nanosensors^61^.

## Methods

### Classical Electromagnetic Calculation

The classical local simulation is performed based on the analytical Generalized Mie theory (GMT), where incident and scattered electric fields are expanded into vector spherical harmonics. The expansion coefficients of incident field are Mie coefficients. The relation between the incidence and multiple scattering is solved by the order of scattering method. Then the scattering cross section can be obtained by the summation of the square of scattering coefficients of different order. The dielectric data of gold are from the work of Johnson and Christy^62^.

### Local Analogue Model

The nonlocal smearing of surface charges in local analogue model (LAM)^44,45^ is mapped into a thin dielectric layer *Δl* on top of the metal surface with permittivity *ε*_*s*_. The shifting of the metal boundary is *Δl*(*ω*) = *ε*_*s*_*λ*(*ω*) *ε*_*M*_(*ω*)/[*ε*_*M*_(*ω*) + 1], where *ε*_*M*_(*ω*) is the permittivity of metal core, and *λ*(*ω*) is the penetration depth of surface charges, which can be written as $\lambda (\omega )=\sqrt{3/5}{V}_{F}/\sqrt{{{\omega }_{P}}^{2}/{\varepsilon }_{M}(\infty )-\omega (\omega +i{\gamma }_{gold})}$. Here, *v*_*F*_, *ω*_*p*_, and *γ*_*gold*_ = 0.09 eV are the Fermi velocity, the bulk plasma frequency, and the damping frequency of gold, respectively. The *Δl* can be obtained by letting the shell dielectric function *ε*_*s*_ equal to the dielectric function of surrounding medium. It should be noted that the modeling thin layer is inside the metal, meaning the gap distance is not affected. By combining LAM with GMT for multiple core/shell particles, scattering spectra incorporating nonlocal effects can be obtained.
